# Effect of the diverse combinations of useful microbes and chemical fertilizers on important traits of potato

**DOI:** 10.1016/j.sjbs.2021.02.070

**Published:** 2021-03-01

**Authors:** Ishan Saini, Prashant Kaushik, Asma A. Al-Huqail, Faheema Khan, Manzer H. Siddiqui

**Affiliations:** a Department of Botany, Kurukshetra University Kurukshetra, Kurukshetra 136119, India; b Instituto de Conservación y Mejora de la Agrodiversidad Valenciana, Universitat Politècnica de València, 46022 Valencia, Spain; c Department of Botany and Microbiology, College of Science, King Saud University, Riyadh 11451, Saudi Arabia

**Keywords:** Arbuscular Mycorrhizal Fungi (AMF), Phosphate Solubilizing Bacteria (PSB), Potato yield, *Nitrosomonas*, *Nitrobacter*, *Bacillus*

## Abstract

The belowground soil environment is an active space for microbes, particularly Arbuscular Mycorrhizal Fungi (AMF) and P hosphate Solubilizing Bacteria (PSB) that can colonize with roots of higher plants. In the present experiment, we evaluated the combination of microbial inoculants with the different doses of urea and superphosphate in a complete randomized block design (CRBD). Three different doses of urea and superphosphate were tested, i.e., recommended dose, 75% of the recommended dose and 125% of the recommended dose, independently and in combination with three microbial groups *viz*. *Glomus mosseae* (AMF), *Bacillus subtilis* (PSB) and Nitrifying microorganisms (*Nitrosomonas* + *Nitrobacter*, NN). Overall, there were 16 treatment combinations used, and studied the number of tubers per plant, the weight of tubers, moisture content, and the number of nodes per tubers which were best in treatment comprising of AMF + PSB + NN + 75% of urea + superphosphate. From our results, it is suggested for the growers to use a lesser quantity of fertilizers from the recommended dose along with some bioinoculants to maintain the soil fertility and also to achieve the yield targets by decreasing the cost of chemical fertilizers.

## 1 Introduction

*Solanum tuberosum* L. is the most extensively cultivated tuberous crop, and it belongs to the family Solanaceae. Potato is a native to Central and South America, and it was introduced in India by Portuguese traders in the early 17th century (Hunziker, 1979, Ayalew, 2014, USDA-NRCS, 2014). Owing to its huge demand, India produces 44 million tonnes with an average of 20.5 tonnes per hectare which occupies 2.13 million hectares of area (Directorate of Economics and Statistics 2017). Although the average yield of potatoes is 20.51 t/ha. There is a difference in cultivation time in North-India and South-India due to agro-ecological diversity and climate change and sometimes season affects the potato growth when temperature increases beyond the optimum (Dua et al., 2013, Haris et al., 2015). Potato needs an extensive supply of fertilizers and has been noted that the soil low in P availability makes potato tubers less developed as P being extremely translocated to the tubers during maturity (Hopkins et al., 2010, Fernandes and Soratto, 2016, Martins et al., 2018). Therefore, P fertilizers are needed for better growth of plants and potato tubers. But, the inappropriate application of fertilizers, drastically disturbs the soil ecosystem and increases heavy metal intoxication (Ozturk et al., 2011, Saini et al., 2019a). Pb, Cd, Ni and Cr are the significant elements present in fertilizers and pesticides can cause biomagnification, out of which Pb and Cd are toxic to potato plant and humans upon consumption even at low concentration (John et al., 2009, Bali et al., 2018, Bali et al., 2019a, Bali et al., 2019b, Jan et al., 2018, Kaur et al., 2017, Kaya et al., 2020a, Kaya et al., 2020b, Kaya et al., 2020c, Ahanger et al., 2020).

Therefore, a better eco-friendly methodology is required; one of the approaches could be the use of mycorrhizal fungi (Srek et al., 2012, Kumar et al., 2015, Abdel Latef et al., 2016, Saini et al., 2017, Begum et al., 2019). By this system, the soil ecosystem can be restored, and nutrient cycling can be stimulated (Collins et al., 2016). The use of arbuscular mycorrhiza fungi (AMF) has proved to enhance the soil organic matter by increasing the biological activity in the plant, nutrient quality and microflora of soil (Pacheco et al., 2021). However, nutrient management is challenging in organic fertilization due to the slow release of nutrients from organic wastes and unable to match per crop demand (Pang and Letey, 2000, Herencia et al., 2007). Thus, biological, and chemical fertilization (NPK) together is a valid consideration in soil nutrient management for improving organically produced crops like potato (Hartz et al., 2000, Collins et al., 2016).

Many factors play a significant role in the dynamics of the symbiosis between the plant and microbes which participate in the exchange of carbon (from plants) and nutrients (from the soil) (Schmidt and Gaudin, 2017). The ability of a plant to adapt in changing environments like the variation in soil texture and various nutrient cycles medicated by microbes are the significant factors for plants to adapt, be more productive and use resources efficiently (Philippot et al., 2013).

One such symbiosis is found between potato and mycorrhizal fungi (Senés-Guerrero, 2014). There are lots of biodiversities, i.e., the totality of all the living organisms, present in the soil rhizosphere (Saini et al., 2019d). Due to the low root -to-shoot ratio, the potato plant is a phosphorus-demanding crop and almost found associated with mycorrhizal fungi (Bhattarai and Mishra, 1984, Dechassa et al., 2003, Mushagalusa et al., 2008). For instance, Senés-Guerrero (2014) have noticed *Funneliformis mosseae* (syn. *Glomus mosseae*), *Rhizophagus irregularis* (syn. *Glomus irregulare*), *Claroideoglomus* sp., *Ambispora*, *Archaeospora* and *Diversispora* were extracted from potato roots. Further, Bharadwaj and colleagues (2007) demonstrated *F*. *mosseae* to be a good colonizer for potato in a greenhouse experiment while *Glomus intraradices* prefer more colonization with potato in arable soils, as reported by Cesaro et al. (2008). Mycorrhizal fungi and Phosphate Solubilizing Bacteria (PSB) during nutrient-deprived conditions release extracellular enzymes that are identified by root exudates released by plant roots in search of P and N (Schimel and Bennett, 2004, Coskun et al., 2017). Several nitrifying bacteria in the agroecosystems also communicate with plant roots for N cycling in conjunction with Arbuscular Mycorrhizal Fungi (AMF) which are interconnected for P cycling through extended mycorrhizal hyphae (Frossard et al., 2000, Jackson et al., 2012, Saini et al., 2019b). For potato, Hannula et al. (2012) showed AMF communities in the potato rhizosphere soil to be affected significantly by the plant growth stage, field site, and year-to-year variation.

Considering these points, an experiment was conducted to elucidate the effect of different concentration of chemical fertilizers (Urea + Superphosphate) along with *Glomus mosseae*, *Bacillus subtilis* (PSB), and Nitrifying microorganisms (*Nitrosomonas* and *Nitrobacter*) on the important traits- related to growth, yield, starch, carbohydrate, nutrient and protein content of *Solanum tuberosum* (Potato) variety Kufri Pukhraj. Kufri Pukhraj was selected for the experiment as it constitutes 33% of total potato farming in India with an area cover of 521,375 Ha (NHB, 2015; Haris et al., 2015). While reviewing other manuscripts, it is speculated that the application of microbes as biofertilizers can enhance the morpho- physiological attributes of Potato (Owen et al., 2015, Saini et al., 2020a). It is also estimated that recommended doses of nitrogen and phosphorus fertilizers can be decreased so that the cost of purchasing fertilizers can also be reduced. This study may further provide trait-level insights and microbial adoption applications that will be useful in shaping future cultivation agendas. Additionally, it will prioritize high-quality variety at a relatively low rate for the producers as well as consumers.

## 2 Materials and methods

The tuber seeds of the variety Kufri Pukhraj were obtained from ICAR-Central Potato Research India, Shimla, India. It is one of the most extensively cultivated potato varieties in the North-Indian plains. The variety has an oval shape with a smooth brown outer cover having some scars and yellowish-cream flesh (Gatto et al., 2018, Pradel et al., 2019).

### 2.1 Experimental setup

The experimentation was carried out in an open field of the Department of Botany, Kurukshetra University, Haryana, India from October 2019 to February 2020 in a complete randomized block design (CRBD) in three replications. A soil culture of *Glomus mosseae* was taken which was propagated as endomycorrhizal species because mycorrhizal fungi are obligate symbionts . Inoculums were arranged from different institutes, and urea was purchased from the local market. The recommended amount for nitrogen fertilizers is 180–240 kg per hectare. The indigenous density of mycorrhizal spores in the experimental site was 35 ± 7 per 10 g soil, which was counted by the gridline intersect method (Adholeya and Gaur 1994).

### 2.2 Formation of starter inoculum

The inoculum of *G*. *mosseae* containing 84–88% colonization (root pieces) and 720–730 AM spores (w/w) was obtained from Forest Pathology Discipline - Forest Protection Division, FRI, Dehradun, India. It was then mass multiplied using sterile sand soil mixture (1:3) and Barley as host for 90 days, in greenhouse conditions. Soil taken for the experiment was evaluated for physical properties like pH: 6.2, organic carbon: 0.56%, total nitrogen: 0.035%, available phosphorus content: 24 ppm, potassium: 41 ppm, assessed by Bandyopadhyay et al. (2012).

*Bacillus subtilis* (MTCC 1305) was procured from the Institute of Microbial Technology (Imtech), Chandigarh, India. *Nitrosomonas* sp. (NCIM 5071) and *Nitrobacter* (NCIM 5062) were taken from the National Collection of Industrial Microorganisms (NCIM), Pune, India. After the procurement of bacteria, they were allowed to be mass-produced without further purification in their respective media. For *B*. *subtilis*, Luria-Bertani broth medium was used containing Tryptone (5 g), Yeast extract (2.5 g) and Sodium chloride (5 g) in 500 ml of distilled water. The medium was incubated in (Biological Oxygen Demand) BOD for 20 min at 37 °C with low background fluorescence, in a 5 l flask (Barns and Weisshaar, 2013). *B*. *subtilis* is a strictly aerobic bacterium, so the flask kept in BOD was having a lid for air passage.

For *Nitrosomonas*, ammonium calcium carbonate medium was used containing Calcium carbonate (7.5 g), Ammonium sulfate (0.5 g), Dipotassium phosphate (1 g), Sodium chloride (3 g), Magnesium sulfate heptahydrate (3 g) and Iron (II) sulfate heptahydrate (0.03 g) in 1 l distilled water. The 500 ml of the medium was used and inoculated at 28 °C for three weeks in BOD (Alexander and Clark, 1965). For *Nitrobacter*, Nitrite-calcium carbonate medium was used containing Calcium carbonate (7.5 g), Potassium nitrite (0.006 g), Sodium chloride (3 g), Magnesium sulfate heptahydrate (3 g), Dipotassium phosphate (1 g) and Iron (II) sulfate heptahydrate (0.03 g) in 1 l distilled water, incubated at 28 °C for three weeks, and only 500 ml was further used (Subba Rao 2009).

### 2.3 Field preparation

**Fig. 1:**
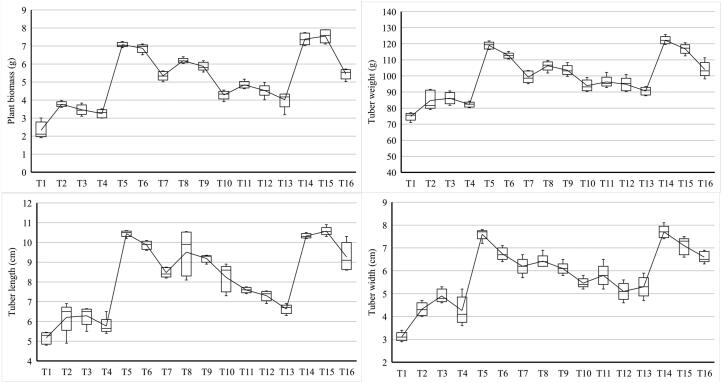
Variation determined among the 16 treatments with different combination of biological and chemical fertilizers in potato for the plant biomass (g), tuber weight (g), tuber length (cm) and tuber width (cm).

First of all, the field of 7 × 9 m low in P content was ploughed at a depth of 15–20 cm thoroughly for proper aeration, and indigenous spores were allowed to sun sanitize by placing a plastic sheet over the ploughed field for 2 days. Then, an almost 3 cm layer of sterilized soil sand mixture was evenly distributed. Farmyard manure and organic waste were added composing 25% of water content, 7.5 pH, 1.05% N, 0.22% P, 0.59% K to make the land loam-clayey measured by Bandyopadhyay et al. (2012). Plant-beds/plots of 1.5 × 1.5 m with 15 cm alleyways were tilled, as shown in Fig. 1. Furrows of 20–30 cm were made on which cut tubers were placed at a depth of 5–7 cm at the centre of the ridge, keeping them at 15–20 cm apart. Each plant-beds were having 3 furrows and 15 plant samples out of which 10 random plants of each treatment were selected for morphological and biochemical analysis after 90 days. Drip irrigation was installed for irrigation.

The first inoculation of *G*. *mosseae* containing 80–82% colonization (Barley root pieces ~ 1 cm) and 410–430 spores (w/w) was given during the planting of tubers in the ridges by putting 10 g soil inoculum under the tubers. The first treatment of *B*. *subtilis*, *Nitrosomonas* and *Nitrobacter* was given by dipping the tubers in respective broth media for 10 min. After 6–7 days when plantlets and adventitious roots (c.a. 10 mm) started appearing, the second treatment of *G*. *mosseae* was given by placing extra soil inoculum around the adventitious roots, to confirm the inoculation. Similarly, the second treatment of *B*. *subtilis*, *Nitrosomonas* and *Nitrobacter* was given by respective sprinkling media on the adventitious roots. This condition was maintained for almost 8–10 days when other irrigation was given. Mycorrhization started after 60–70 days of inoculum and was confirmed as well as quantified by Trypan blue staining (Phillips and Hayman 1970; INVAM 2017). The percentage of root colonized was determined as the proportion of root colonized over the total number of roots examined and multiplying the figure by 100 (Giovannetti and Mosse 1980).

### 2.4 Experimental design

Four different combinations of microbial inoculums were selected, and 3 concentrations of urea (U) and superphosphate (SP) treatment were used a) lower the amount of the recommended dose, i.e., 75% (US_P75_), b) recommended cultivation dose, i.e., 100% (USP_100_) and c) higher the amount of the recommended dose, i.e., 125% (USP_125_). The optimum N fertilizer and P fertilizer doses for potato crops are 200–300 kg ha^−1^ and 50–150 kg ha^−1^, respectively (Lang et al., 1999, George and Ed, 2011). For the experiment, we used 250 kg N ha^−1^ and 100 kg P ha^−1^ as recommended doses or 100% doses. The control plot was not having any added bioinoculants or fertilizer doses. Microbial inoculums and urea treatment/s were added as independent as well as mixed in different concentrations, as shown in Table 1. Whereas, the list of sixteen different treatment combinations of chemical fertilizers and the microbial inoculum is represented in Table 2.

**Table 1:** Showing treatments with three different concentrations of urea (U) and superphosphate (SP) on *S. tuberosum*.

Chemical Fertilizers	Microbial inoculum			
	G_m_	B_s_	NN	G_m_ + B_s_ + NN
US_P75_	G_m_ + US_P75_	B_s_ + US_P75_	NN + USP_75_	G_m_ + B_s_ + NN + USP_75_
US_P100_	G_m_ + US_P100_	B_s_ + US_P100_	NN + US_P100_	G_m_ + B_s_ + NN + USP_100_
US_P125_	G_m_ + US_P125_	B_s_ + US_P125_	NN + US_P125_	G_m_ + B_s_ + NN + USP_125_

Gm- *Glomus mosseae*; Bs- *Bacillus subtilis*; NN– Nitrosomonas + Nitrobacter USP - Urea + Superphosphate; USP75 − 75% recommended dose; U¬SP100 ¬¬- 100%/Actual recommended dose; USP125 − 125% recommended dose.

**Table 2:** The following sixteen treatments (Tt) were studied with the combination of bioinoculants and the chemical fertilizers for *S. tuberosum*.

Code	Treatments*
Tt1	C
Tt2	US_P75_
Tt3	US_P100_
Tt4	US_P125_
Tt5	G_m_ + US_P75_
Tt6	G_m_ + US_P100_
Tt7	G_m_ + US_P125_
Tt8	B_s_ + US_P75_
Tt9	B_s_ + US_P100_
Tt10	B_s_ + US_P125_
Tt11	NN + US_P75_
Tt12	NN + US_P100_
Tt13	NN + US_P125_
Tt14	G_m_ + B_s_ + NN + US_P75_
Tt15	G_m_ + B_s_ + NN + US_P100_
Tt16	G_m_ + B_s_ + NN + US_P125_

* Where, G_m_- *Glomus mosseae*; B_s_- *Bacillus subtilis*; NN– *Nitrosomonas* + *Nitrobacter*; U- Urea; S_P_- Superphosphate; US_P75_- 75% of Recommended dose; US_P100_- 100% of Recommended dose/Actual; US_P125_- 125% of Recommended dose.

### 2.5 Morphological characterization

Out of fifteen plants in each plot, five plants were selected randomly for examination. Morphological characters and yield parameters like plant biomass, tuber number and weight, moisture content, number of nodes per tuber were measured, considering Esendal, 1990, Högy and Fangmeier, 2009. Plant biomass was calculated by subtracting fresh weight with dry weight (oven-dry at 55 °C for 2 days) of harvested plants and expressed in grams (g). The number of potato tubers was counted upon harvesting, and five healthiest tubers were selected for the accounting number of nodes and weight. The weighed potatoes were kept for oven-dry (60 °C for 3 days) and noted the dry weight. After this moisture content in percentage was calculated by the given formula.

$$\mathrm{Moisturecontent}=\frac{\left(Freshweight-DryWeight\right)}{\mathrm{Freshweight}}\times 100$$

The tuber size (lengthwise and widthwise) was measured via a measuring scale. Firstly, by keeping the potato on the paper and two lines each was marked lengthwise plus breadthwise, then these lines' gaps were measured. Muslin cloth was removed during maturity, which was earlier placed to cover the crop.

### 2.6 Biochemical characterization

Total carbohydrate and starch content, nutrient and bio-physiochemical processes like total P and N content, shoot phosphatase activity, and total chlorophyll was determined. Total carbohydrate (containing free sugars and polysaccharides) was estimated in mg per 100 mg fresh weight by the ‘Anthrone method’ using glucose, acid hydrolysis by HCN, and Anthrone Reagent, taking absorption at 630 nm. Carbohydrate content present in 100 mg of the potato sample was calculated by mg of glucose divided by the volume of the testing sample and multiplying by 100 (Hedge and Hofreiter 1962). In contrast, the starch content was similarly determined by the ‘Anthrone method’ in mg per 100 mg fresh weight using glucose, ice-cold acid hydrolysis by sulphuric acid, perchloric acid and ethanol, taking the absorption at 630 nm (Hedge and Hofreiter, 1962, Thayumanavan and Sadasivam, 1984). Chlorophyll content was estimated by ‘CL-01 (Hansateh) Chlorophyll Content Meter,’ Hansateh Instruments Ltd., Norfolk, United Kingdom, that uses dual-wavelength optical absorbance of 620 and 940 nm.

While, total N content in percent (nitrate, nitrite, heterocyclic N, nitrogen-containing amino acid and protein) was calculated by the ‘Automated combustion method’ using EDTA and glycine p-toluenesulfonate. All the absorptions were taken by a UV–Vis. Spectrophotometer (Specord- 205 Analytik Jena AG, Jena, Germany). AMF number was counted by ‘Gridline Intersect method’ using Whatman filter paper no 1 and AMF colonization quantification was done by Phillips and Hayman staining method (1970) followed by ‘Giovannetti and Mosse’ (1980), both analyzed under Lab Digital Trinocular Compound LED Microscope (Omax 40X-2500X).

### 2.7 Statistical analyses

Analysis of Variance (ANOVA) was conducted to detect the differences among means of each treatment using the SPSS (11.5 version) software package (Nie et al. 1975). Each mean was exposed to one-way ANOVA that examined the effect of AMF-inoculation. The results of the experiment were analyzed for studying parameters between control and mycorrhizal-inoculated plants, and the significance of differences was calculated using least significant differences (LSD) P < 0.05 according to Steel and Torrie (1980).

## 3 Results

AMF showed symbiosis with almost all vegetable crops and is beneficial for increasing water and immobile nutrients absorption through positively stimulating the rhizospheric expansions by 67–76% (Table 3, Table 4). All the treated plants showed a positive effect over control except the increased dose treatment of urea and superphosphate (Fig. 1). Yet, it was noticed that on inoculating microbes, this negative effect was somewhat controlled (Table 3, Table 4). The number of the combination was found to be best for different parameters which showed that by decreasing the quantity of fertilizers used in the field by 25% and addition of microbial treatments, the growth and yield were enhanced (Fig. 1). Significant differences (p ≤ 0.05) were determined for the yield and morphological traits among 16 different treatments comprising of beneficial soil microbes and the chemical fertilizers applied to the potato (Table 3). Plant biomass was increased by 204%, 218%, and 225% in the Tt5, i.e., the treatment with 75% of the recommended dose urea and superphosphate, Tt14 which is a treatment with the microbial consortium and 75% of the recommended dose of urea and superphosphate, and in Tt15 comprising of microbial consortium and full dosage of chemical fertilizers (Table 3). Tuber weight was increased by 56.29%, 59.12% and 63.46% in the treatments Tt15, Tt5 and Tt14 respectively (Table 3). Whereas tuber length was increased by 99.80%, 102.12%, and 104.44% in the treatments Tt 14, Tt5 and Tt15 and tuber width was increased in the treatments Tt15, Tt5, and Tt14 by 129.48%, 143.58% and 147.75% (Table 3). The number of tubers per plant were increased by 121.73%,126.08% and 134.78% in the treatments Tt15, Tt5 and Tt14. Moreover, an increment of up to 83.33% was reported for the trait number of nodes per tuber in the treatments Tt5, Tt6 and Tt14 (Table 3).

**Table 3:** Effect of different concentrations of urea + Superphosphate along with bioinoculants on morphological and yield of *S. tuberosum*.

Treatments	Plant biomass (g)	Tuber weight (g)	Tuber length (cm)	Tuber width (cm)	Number of tubers per plant	Number of nodes per tuber
Tt1	2.32 ± 0.454 l*	74.77 ± 2.38j	5.17 ± 0.29i	3.12 ± 0.19 g	4.60 ± 0.54 g	4.80 ± 0.83e
Tt2	3.76 ± 0.142ij	84.73 ± 5.93i	6.21 ± 0.77 fg	4.32 ± 0.28f	5.40 ± 1.35 fg	5.60 ± 0.54d
Tt3	3.46 ± 0.279jk	86.09 ± 3.58i	6.29 ± 0.42 fg	4.89 ± 0.29ef	5.80 ± 0.82f	5.80 ± 0.83d
Tt4	3.25 ± 0.229 k	82.25 ± 1.62i	5.77 ± 0.42gh	4.26 ± 0.61f	4.80 ± 0.44 fg	5.20 ± 1.09d
Tt5	7.07 ± 0.133bc	118.98 ± 2.225b	10.45 ± 0.15ab	7.60 ± 0.23ab	10.40 ± 0.55ab	8.80 ± 0.44a
Tt6	6.87 ± 0.236c	112.62 ± 1.74c	9.86 ± .21bc	6.74 ± 0.27 cd	9.60 ± 1.14bc	8.80 ± 0.83a
Tt7	5.34 ± 0.247e	99.27 ± 3.69ef	8.47 ± 0.24d	6.20 ± 0.36 cd	8.60 ± 0.56de	7.60 ± 0.89bc
Tt8	6.18 ± 0.141d	106.31 ± 2.93d	9.51 ± 1.14c	6.42 ± 0.28 cd	9.40 ± 1.34 cd	8.40 ± 1.14ab
Tt9	5.86 ± 0.238d	103.55 ± 3.16de	9.19 ± 0.18c	6.09 ± 0.25 cd	9.20 ± 0.85c	8.60 ± 0.89ab
Tt10	4.28 ± 0.241hi	93.94 ± 3.59gh	8.23 ± 0.69d	5.46 ± 0.21ef	8.40 ± 0.52de	7.20 ± 1.09bc
Tt11	4.84 ± 0.204f	96.28 ± 3.55 fg	7.59 ± 0.14e	5.80 ± 0.47de	8.20 ± 0.83de	7.60 ± 0.89bc
Tt12	4.53 ± 0.344gh	94.81 ± 4.28gh	7.31 ± 0.26e	5.11 ± 0.38ef	7.60 ± 0.89e	6.80 ± 0.83c
Tt13	4.01 ± 0.478hi	90.65 ± 2.47 h	6.63 ± 0.23ef	5.29 ± 0.44ef	7.80 ± 0.84e	8.20 ± 0.83ab
Tt14	7.38 ± 0.326ab	122.22 ± 2.43a	10.33 ± 0.12ab	7.73 ± 0.27a	10.80 ± 0.83a	8.80 ± 0.44a
Tt15	7.54 ± 0.365a	116.86 ± 3.01bc	10.57 ± 0.21a	7.16 ± 0.38bc	10.20 ± 1.09ab	8.60 ± 0.54ab
Tt16	5.45 ± 0.283e	104.03 ± 4.82d	9.27 ± 0.72c	6.63 ± 0.24 cd	8.80 ± 0.44cde	7.80 ± 0.44bc
ANOVA (F 15, 64)	151.24	83.51	68.11	4.61	26.24	14.04
LSD (P ≤ 0.05)	0.36	4.29	0.61	1.58	1.09	1.03

* ±- Standard deviation; values in a column followed by the same letter are not significantly different; p ≤ 0.05- LSD (least significant difference test).

**Table 4:** Effect of different concentrations of urea + Superphosphate along with bioinoculants on bio-physiological and colonization characters of *S. tuberosum*.

Treatments	Total carbohydrates (mg/100 mg FW)	Starch content (mg/100 mg FW)	Total N content (%)	Moisture content (%)	Chlorophyll (mg g-1 FW)	AMF no.	AMF colonization %
Tt1	14.31 ± 1.05i*	18.14 ± 1.28i	0.33 ± 0.10i	54.08 ± 0.81o	20.65 ± 1.13 k	39.00 ± 3.53i	22.92 ± 2.72 g
Tt2	17.22 ± 1.18 h	21.34 ± 1.79 h	0.44 ± 0.03 h	63.14 ± 0.28 l	23.95 ± 2.09ij	45.00 ± 4.30 h	31.08 ± 3.81 fg
Tt3	17.56 ± 0.65 fg	21.82 ± 0.51 h	0.48 ± 0.48 h	62.53 ± 0.62 *m*	24.13 ± 0.86ij	49.00 ± 2.91gh	33.02 ± 4.56 fg
Tt4	17.01 ± 0.68 h	21.07 ± 1.66 h	0.46 ± 0.08 h	61.38 ± 0.69*n*	23.02 ± 2.47j	40.00 ± 2.23i	32.11 ± 4.65 fg
Tt5	21.26 ± 1.16ab	30.29 ± 0.86ab	0.71 ± 0.09gh	84.91 ± 0.57b	37.55 ± 2.67ab	230.00 ± 7.10b	70.13 ± 3.30b
Tt6	20.25 ± 0.98bc	29.13 ± 0.79b	0.76 ± 0.11 fg	77.02 ± 0.37d	32.44 ± 1.13de	222.00 ± 8.63c	65.09 ± 1.52bc
Tt7	18.33 ± 0.52 fg	25.21 ± 0.71de	0.75 ± 0.09 fg	68.01 ± 0.63 h	33.33 ± 1.73de	107.00 ± 4.94e	58.12 ± 3.65de
Tt8	19.63 ± 1.06 cd	26.51 ± 1.18c	0.51 ± 0.12gh	74.32 ± 0.72e	31.48 ± 1.27e	115.00 ± 3.60d	63.75 ± 3.66 cd
Tt9	19.02 ± 1.14de	25.76 ± 1.04de	0.54 ± 0.11gh	71.13 ± 0.49f	31.07 ± 2.09e	110.00 ± 6.20de	61.14 ± 5.92de
Tt10	17.43 ± 0.72 g	23.62 ± 0.52 fg	0.52 ± 0.17gh	67.16 ± 0.39i	27.52 ± 1.59 fg	97.00 ± 5.24f	57.15 ± 7.67de
Tt11	18.06 ± 0.85 fg	24.74 ± 1.14ef	0.94 ± 0.17ef	66.13 ± 0.31j	28.74 ± 1.27f	49.00 ± 3.16 g	41.61 ± 6.65ef
Tt12	17.82 ± 0.51 fg	24.32 ± 1.63ef	1.18 ± 0.29d	65.15 ± 0.38 k	26.48 ± 1.42 h	44.00 ± 3.53j	45.81 ± 5.71ef
Tt13	17.22 ± 1.01 g	22.52 ± 1.13gh	1.06 ± 0.16de	64.21 ± 0.52 l	25.71 ± 1.65hi	39.00 ± 4.30i	45.33 ± 3.87ef
Tt14	21.79 ± 0.97a	31.64 ± 1.32a	1.45 ± 0.25c	88.11 ± 1.41a	39.15 ± 1.18a	245.00 ± 5.52a	79.39 ± 4.55a
Tt15	20.54 ± 0.51bc	29.76 ± 0.92b	1.51 ± 0.32a	81.16 ± 0.64c	35.44 ± 1.38bc	225.00 ± 7.44bc	67.24 ± 4.81bc
Tt16	18.77 ± 0.92ef	26.34 ± 0.56c	1.48 ± 0.214b	70.03 ± 0.37 g	34.44 ± 1.68 cd	112.00 ± 4.30de	66.53 ± 4.50bc
ANOVA (F 15, 64)	21.57	54.09	26.88	1027.46	53.69	1141.81	63.21
LSD (P ≤ 0.05)	1.14	1.44	0.21	0.80	2.12	6.46‬	3.19

* ±- Standard deviation; values in a column followed by the same letter are not significantly different; p ≤ 0.05- LSD (least significant difference test.

Significant differences (p ≤ 0.05) were determined for the tuber biochemical composition traits (Table 4). Total carbohydrates increased by 43.53% in Tt15, 48.56% in Tt5 and by 52.27% in Tt14. Moreover, in the same direction, the best three treatment with the highest starch content were Tt15, Tt5 and Tt14 in these treatments the starch content was increased by 64.05%, 66.97% and 74.42％ respectively (Table 4). Total N content was highest in the treatments Tt14, Tt16 and Tt15 with an increase of 339.39%, 348.48% and 357.57% respectively. Moisture content was increased in the treatments Tt15, Tt5 and Tt14 by 50.07%, 57% and 62.92%. Chlorophyll content was significantly improved in treatments Tt15, Tt5 and Tt14 by 71.62%, 81.84%, and 89.58% (Table 4). AMF number was recorded highest in the treatments Tt15, Tt5, and Tt14. In the same direction, AMF colonization % was increased by 193.36%, 205.97% and 246.37% in the treatments Tt15, Tt5 and Tt14, respectively (Table 4). A similar trend was determined for the starch content (mg/100 mg FW), which was also twice the starch content in the control treatment tuber content (Table 4).

## 4 Discussion

The rhizospheric soil is considered as hotspots for microbial activity and when extra microbial inoculation was added to the potato (Rana et al., 2020). This inoculation in combination with urea and superphosphate proved to be better for potato growth and yield. *G*. *mosseae*, *B*. *subtilis* and nitrifying bacteria can potentially interact synergistically as a consequence, the root system lives in mutualistic harmony (Ordoñez et al., 2016). This interaction influence the nutrient absorption, especially phosphorus (Saini et al., 2020b). Potato is high-quality nutrients crop. It has a high glycemic index and easy to digest. Potato has relatively small root architecture, and that’s why the absorption of nutrients is somewhat problematic, particularly P, which is important for potato tuber formation. The effect of bioinoculants after 90 days on the microbial symbiosis, soil profile and potato production resulted in positive observation as expected. As we know, in natural soil there are millions of microbial colonies playing a profound ecological role in organic material recycling and biogeochemical cycling processes, helpful in plant growth and development (Sangwan et al., 2012, Rana et al., 2020). For instance, plant biomass fertilized with chemical fertilizers and microbial inoculation had shown enhanced effect because the microbial population makes the absorption of the nutrients easy as compared to those which were not inoculated with microbes, as this favours large aggregation of oligotrophic bacteria like *Verrucomicrobia*, *Acidobacteria*, *Planctomycetes*, etc (Ramírez-Villanueva et al., 2015). This ought to be the reason why potato tubers inoculated by microbes showed higher biomass and size as compared to control. Moreover, the addition of litter/organic matter along with microbial inoculants and agrochemicals in the field via bioturbation and/or leaching increased microbial activity in the soil which stabilizes the soil bio-physio-chemical composition and decrease the polyphenol activity (Kaneda et al., 2013, Frouz, 2018). This practice creates a long-lasting effect on the soil and plant growth, and this might be the reason for bigger size potato tuber. The bigger size of the tuber corresponds to a large number of eyes and the weight of the potato. Therefore, AMF, PSB and NN can interact synergistically when extra P and N are supplied. This confirms our findings why 75% of urea and superphosphate along with microbial inoculation showed the best growth. Furthermore, Yanardağ et al. (2013) also reported the use of bio-farming, which helps in organic matter mineralization, nutrient stabilization and immobilization processes, hence the quality of soil enriches, and yield was enhanced. In addition to this, phytohormones like auxin, gibberellin, etc. production is stimulated when plants are inoculated by microbes, which further add clarification of increased plant biomass and tuber weight (Glick, 2012, Umair et al., 2018). AMF and other microbes can also assist the potato tuber in acquiring nitrogen present in the soil in the form of nitrates and ammonium ions by increasing nitrate reductase and glutamine synthase (Shuab et al., 2017). An increase in phytohormones concentration effectively influences the development of the root system, which can easily absorb water and nutrients for better growth of potato (Chaiharn 2011). All these factors are inter-dependent and cannot be explained separately. Yasmin et al. (2020) reported similar results, an increase in sweet potato yield per plant by inoculating *Bacillus*, *Klebsiella*, *Azospirillum* and *Erwinia* which can also be regarded as Plant Growth-Promoting Rhizobacteria (PGPR) along with three concentration of N fertilizers. Hijri (2016) also demonstrated the benefits of microbes (AMF: *Glomus intraradices*) on potato crop yield in field conditions. Previous Study by Ceballos et al. (2013) have reported that large-scale production of potato fortified with AMF is feasible, and also this allows a reduction in chemical fertilization by 25–50%, this also supports our finding.

In the investigation, it was noted that inoculation on potato tubers and/or roots helps to increase the magnitude of colonization. The PSB can also be called mycorrhizal-helper bacteria that solubilize the phosphate from the inorganic or organic compound for the easy fascination of water and nutrients by extraradical hyphae of AMF that absorb 100-folds faster than normal roots, as mentioned above (Bitterlich and Franken, 2016, Saini et al., 2019a). And, this is the reason why moisture content in our findings increased. The increased range was 67–76%, which was similar to 72–87% of Ritter et al. (2008) and 63–87% of Burlingame et al. (2009). The combination of nitrifying bacteria, PSB and AMF for potato may facilitate efficient uptake of N and P without even using chemical fertilizers (Ngakou et al., 2006, Jackson et al., 2012).

T reatments having only urea and superphosphate concentrations, developed diffuse and thick roots that may intake fewer nutrients, but when they are inoculated with AMF and PSB roots showed thinner and longer root architecture for absorbing nutrients (Ordoñez et al., 2016). There is a direct connection between chlorophyll, carbohydrate, and starch content by photosynthesis. More Mg, N, P, Mn, Cl, etc. absorption will have higher rate photosynthesis and so the starch and carbohydrate content (Srek et al., 2012, Shuab et al., 2017). As discussed, many phytohormones are stimulated by microbial inoculation, besides that increase in chlorophyll activity, chlorophyll number, stomatal conductance and stomata number was also observed (Boldt et al., 2011, Arumugam et al., 2010). That further approves our results in the present experiment. Besides, the siderophores (iron-chelating agent) may also get increased which is beneficial for photosynthesis and respiration, and that’s total starch, and carbohydrate content got increased in microbial plus chemical fertilizers treatment (Kobayashi and Nishizawa 2012). Nurbaity et al. (2016) conducted a greenhouse experiment and concluded that the application of *Glomus* and *Pseudomonas* reduced the use of NPK by up to 50% on the potato plant. Whereas, they also found that increasing fertilizer quantity had a similar effect on plant yield and NPK uptake. Hijri (2016) in Europe and North America revealed that application of *R*. *irregularis* in potato produced a marketable yield. On the other hand, Loján et al. (2017) concluded that in Ecuadorian Andes while inoculating *Rhizophagus irregularis*, there is no significant increase in potato tuber growth. This highlighted that AM biogeography also play important role in colonization. Loján et al. (2017) also added that inoculation technique should be proper and AMF interact with indigenous background AM fungi and soil nutrient status that also affect the crop growth significantly. But, application of microbial inoculant in our experiment with lesser amount of urea and superphosphate showed emphatic response. The benefits of endomycorhhiza was also concluded by Bitterlich and co-workers (2018) as nutritional and bioprotective (abiotic and biotic) benefits contributing crop yield in agriculture system. That’s why it is important to note that reducing the number of fertilizers and adding some bioinoculants effectively improve the quality of the crop.

## 5 Conclusion

The potato crop is a valuable food commodity that needs to be cultivated with full care and eco-friendly N and P fertilizer inputs. A critical next step is about observing the various changes occurring in soil concerning their interaction with microbes at different points of crop growth in a season. From the results, it is suggested that rhizospheric microbes form strong interaction with roots for nutrient and water uptake and revealed that they influence water use efficiency, nutrient cycling and yield. Microbial inoculation allows the formation of useful secondary metabolites which make the plant resistant to pathogenic and pests attack. With all the parameters studied, this can be determined that AMF (*G*. *mosseae*), PSB (*B*. *subtilis*) and nitrogen fertilizer inoculants (Nitrosomonas + Nitrobacter) can significantly be used for crop growth and yield. The present experiment showed the different inoculation pattern differs in several morphological and biochemical aspects. The consortium treatment *G*. *mosseae*, *B*. *subtilis*, *Nitrosomonas* + *Nitrobacter* with 75% of recommended doses of urea and superphosphate (G_m_ + B_s_ + NN + US_P75_) is the overall best treatment. Therefore, further studies are needed to minimize the agrochemical compounds to lessen the soil damage.

## Declarations

Conflicts of interest/Competing interests: Authors declare that no conflict of interest exists.

Ethics approval: Not Applicable.

Consent to participate: All authors consent to participate in this manuscript.

Consent for publication: All authors consent to publish this manuscript in Saudi Journal of Biological Science.

Availability of data and material: Data will be available on request to corresponding or first author.

Code availability: Not Applicable.

Authors' contributions: P.K, AAA, MHS designed the study. Experiments were performed by I.S., and P.K. Data were analyzed by PK and FK. The manuscript was written by I.S. and P.K. PK, AAA and MHS revised the final draft.

## Declaration of Competing Interest

The authors declare that they have no known competing financial interests or personal relationships that could have appeared to influence the work reported in this paper.
